# Dynamics of the Type I Interferon Response During Immunosuppressive Therapy in Rheumatoid Arthritis

**DOI:** 10.3389/fimmu.2019.00902

**Published:** 2019-04-24

**Authors:** Tamarah D. de Jong, Tanja Snoek, Elise Mantel, Conny J. van der Laken, Ronald F. van Vollenhoven, Willem F. Lems

**Affiliations:** Amsterdam UMC, Vrije Universiteit Amsterdam, Rheumatology, Amsterdam Rheumatology and Immunology Center, Amsterdam, Netherlands

**Keywords:** rheumatoid arthritis, interferon, interferon response, biomarker, immunosuppression

## Abstract

**Objective:** The type I interferon (IFN) response in rheumatoid arthritis (RA) has been extensively studied in relation to therapy with biological DMARDs (bDMARDs). However, the effect of conventional synthetic (cs)DMARDs and glucocorticoids (GCs) on IFN response gene (IRG) expression remains largely unknown, even though csDMARDS are used throughout all disease phases, including simultaneously with biologic therapy. This study was aimed to determine the dynamics of IFN response upon immunosuppressive treatment.

**Methods:** Whole blood was collected in PAXgene tubes from 35 RA patients who received either COBRA therapy (combination of prednisone, initially 60 mg, methotrexate and sulfasalazine) (*n* = 14) or COBRA-light therapy (prednisone, initially 30 mg, and methotrexate) (*n* = 21). Expression of 10 IRGs was determined by real-time PCR at baseline (T0), after 4 weeks (T4), and 13 weeks (T13) of treatment. IRG selection was based on the differential presence of transcription factor binding sites (TFBS), in order to study the therapy effect on different pathway components involved in IFN signaling.

**Results:** Seven of the 10 IRGs displayed significant changes during treatment (*p* ≤ 0.016). These 7 IRGs all displayed a particularly pronounced decrease between T0 and T4 (≥1.6-fold, *p* ≤ 0.0059). The differences between IRG sensitivity to the treatment appeared related to the presence of TFBS for STAT1 and IRF proteins within the genes. The extent of the decreases between T0 and T4 was similar for the COBRA- and COBRA-light-treated group, despite the differences in drug combination and doses in those groups. Between T4 and T13, however, IRG expression in the COBRA-light-treated group displayed a significant increase, whereas it remained stable or decreased even further in most COBRA-treated patients (comparison of mean fold changes, *p* = 0.011). A significant association between IRG dynamics and clinical response to therapy was not detected.

**Conclusions:** Immunosuppressive treatment with csDMARDs, in this case a combination of prednisolone, methotrexate and sulfasalazine, substantially downregulates the IFN response in RA patients. The dynamics of this downregulation were partly dependent on the presence of TFBS within the IRGs and the combination and dosages of agents, but they were irrespective of the clinical response to therapy.

## Introduction

Early treatment of rheumatoid arthritis (RA) has proven effective in decreasing disease activity and limiting joint damage (1, 2). One treatment strategy which has shown effectiveness in early RA is COBRA (Dutch acronym for COmbinatietherapy Bij Reumatoïde Arthritis), which is a step-down strategy consisting of initial high dose prednisolone (60 mg per day), methotrexate (MTX) and sulfasalazine (SSZ). Due to rheumatologists' concerns with respect to the high initial prednisolone dose and the complexity of the drug schedule, COBRA-light strategy was introduced, which consists of a lower initial prednisolone dose (30 mg/day), combined with increasing doses of MTX (10–25 mg in 9 weeks) and no SSZ. The two strategies have shown to be similarly effective (3–5).

The use of glucocorticoids (GCs) such as prednisolone and conventional synthetic disease-modifying anti-rheumatic drugs (csDMARDs) such as MTX and SSZ is not restricted to early disease. In fact, these therapies are used throughout all phases of the disease, either as monotherapy or in combination, including simultaneously with biologic therapy (6).

With regard to biologic therapy, we have previously demonstrated that the predictive performance of the type I interferon (IFN) response gene set for non-response to rituximab was impaired when patients were using prednisolone at the moment of blood collection (7). Besides rituximab, IFN response gene (IRG) expression has also been described as a predictor for other bDMARDS such as anti-TNF agents and tocilizumab, and RA onset (8–11).

However, studies on the potential influence of csDMARD and GC (co-)medication yet remain scarce. Insight into the effect of these therapies on the IFN response, as well as the potential relation between IRG expression and the clinical response to csDMARD and GC therapy, are highly relevant in order to further understand the role of the IFN response in RA.

*In vitro* studies have shown that GC signaling could inhibit type I IFN signaling by competition for the same intracellular signaling components, i.e., the IFN regulatory factors (IRFs) (12, 13) and by inhibition of the transcription factor STAT1 (14). Accordingly, we have observed that RA patients who were treated with the GC prednisolone indeed displayed lower IRG expression compared to patients who had not received this treatment (7, 15). Although this decrease was not observed with methotrexate (MTX) use and appeared dependent on prednisolone dose, a causal relation could not be established due to the cross-sectional nature of the study. Moreover, since the study was performed in patients who were about to start on biologic therapy, hence who no longer benefitted from the csDMARD and GC therapies, an analysis in relation to clinical response to these therapies could not be made. The present study was focused on exploration of the IFN response during COBRA and COBRA-LIGHT therapy in RA. The sample collection within the COBRA and COBRA-light cohorts enabled us to investigate this in a longitudinal manner and additionally examine the potential relation with clinical response.

## Methods

### Patients and Treatment

All patients in the current study participated in the COBRA-light study, a randomized, open, multicenter trial comparing two treatment schedules for the treatment of early RA (http://www.controlled-trials.com; ISRCTN55552928). Details of that study have been reported previously (3). In short, DMARD-naïve Dutch patients with recent-onset RA according to the 1987 revised American College of Rheumatology criteria (16) were included and randomized to the COBRA-light or COBRA strategy. Whereas, COBRA therapy consists of initially high-dose prednisolone (60 mg/day) combined with sulfasalazine (SSZ) and low-dose methotrexate (MTX) (7.5 mg/week), COBRA-light consists of a lower initial prednisolone dose (30 mg/day) but a higher starting dose of MTX (10 mg/week) and no SSZ.

For this study, 36 patients were selected based on availability of PAXgene tubes at baseline (T0), after 4 weeks (T4) and 13 weeks (T13) at the Amsterdam Rheumatology and Immunology Center, location Reade, Amsterdam, The Netherlands. Fifteen patients received COBRA therapy and 21 patients received COBRA-light therapy. Therapy response was defined as a Disease Activity Score in 44 joints (DAS) ≤ 2.4 after 26 weeks of treatment. Additionally, the change in DAS (ΔDAS) after 13 weeks and 26 weeks was also assessed.

This study was approved by the medical ethics committee of VU University Medical Center and Reade, Amsterdam, The Netherlands, and informed consent was obtained from all donors.

### RNA Isolation and cDNA Synthesis

From each donor, blood was collected into a PAXgene tube (PreAnalytiX GmbH) at baseline and after 4 weeks and 13 weeks of treatment. The PAXgene tubes were stored at −20°C until further processing. After overnight thawing at room temperature, total RNA was isolated using the PAXgene Blood RNA kit (PreAnalytiX GmbH) according to the manufacturer's instructions. Total RNA concentration was measured using the Nanodrop spectrophotometer (ThermoFisher Scientific Inc.). From each sample, 250 ng RNA was reverse-transcribed into cDNA using a Revertaid H-minus cDNA synthesis kit (ThermoFisher Scientific Inc.).

### Interferon Response Gene Selection and Real-Time PCR

Because GCs have been demonstrated to inhibit the IFN response *in vitro* via interaction with specific signaling components such as IFN regulatory factors (IRFs) (12, 13) and STAT1 protein (14), three IFN response genes (IRGs) were selected for the presence of specific transcription factor binding sites (TFBS). Thereto, all 45 IRGs that were previously described to be part of the IFN signature in RA (17), were submitted to the Transfac algorithm available from Interferome (http://interferome.its.monash.edu.au), an online database of IRGs (18). As shown in Table 1, *IL1RN* only contained a binding site for the transcription factors STAT3, *IFITM1* only for NFκB and *IFI6* only for IRF-proteins, such as IRF7, IRF8, and IRF9, which binds the IFN responsive element (ISRE). In addition, *RSAD2, MX1*, and *IFI44L* were taken along as positive controls because of their known well-detectability (9, 15). To confirm our initial observations, four additional genes were included based on the presence of certain TFBS (see Table 1). Real-time PCR was performed using Taqman gene expression assays and ABI Prism 7500 HT Sequence Detection System (Thermo Fisher Scientific Inc.), according to the manufacturer's protocols. Gene expression values were calculated relative to a standard curve and normalized to the average expression of two housekeeping genes: *18S rRNA* and *HPRT*.

**Table 1:** IFN response gene selection.

**Genes**	**Transcription factor binding sites**				**Reason for selection**
	**IRF proteins**	**STAT1**	**STAT3**	**NFκB**	
**INITIAL GENE SELECTION**					
*IFI44L*	IRF7, IRF8	X	X	–	Technical control
*IFI6*	IRF7, IRF8, ISRE	–	–	–	IRF-specific
*IFITM1*	–	–	–	X	NFκB-specific
*IL1RN*	–	–	X	–	STAT-specific
*MX1*	IRF7, ISRE	X	X	–	Technical control
*RSAD2*	IRF7	–	X	–	Technical control
**ADDITIONAL SELECTION**					
*HERC5*	IRF7, ISRE	–	–	–	IRF-specific
*IFITM2*	–	–	X	X	IRF- and STAT1-lacking
*LY6E*	–	X	X	X	IRF-lacking
*SERPING1*	–	X	X	X	IRF-lacking

*IRGs that contained a binding site for only one type of transcription factor were selected. Additionally, three other genes were included as technical controls*.

*“X” indicates that the gene contains a binding site for that transcription factor, whereas “–” indicates absence of the TFBS in that gene. ISRE; IFN stimulated response element, binding site of the ISGF3 complex which consists of STAT1, STAT2, and IRF9. Binding is IRF9-dependent, hence this is considered an IRF-specific binding site*.

### Statistical Analysis

One patient was not included in the analyses as the RNA yield of its T4 sample was not sufficient for further measurements. Statistical analyses were performed using IBM SPSS Statistics 22. Data normality was checked according to Shapiro-Wilk test, with a normal distribution if *p* > 0.05. Because most data were not normally distributed, non-parametric tests were used for most comparisons. Longitudinal changes in IRG expression during treatment were tested using Friedman tests, followed by Wilcoxon signed ranks test. The comparisons of COBRA and COBRA-light therapy and responders and non-responders were performed using Mann-Whitney U test. Correlations between IRG expression and ΔDAS were assessed using Spearman correlation and correlations between IRG expression and ^2^log-transformed CRP and ESR ratios were assessed using Pearson correlation. *P* < 0.05 were considered statistically significant.

## Results

### Patient Characteristics

Demographic and clinical data are shown in Table 2. No significant differences were observed in clinical characteristics between the COBRA and the COBRA-light group. After 26 weeks, the COBRA-light group displayed a higher DAS value and a lower percentage of patients with DAS values below 2.4. However, these differences did not reach significance, neither at later time points (data not shown, *p* ≥ 0.45), which is in line with previously demonstrated non-inferiority of COBRA-light versus COBRA therapy (3–5).

**Table 2:** Cohort characteristics of the COBRA and COBRA-light groups.

	**All patients**	**COBRA group**	**COBRA-light group**
*N*	35	14	21
Age, years, median (IQR)	54 (45–60)	56 (44–61)	54 (45–59)
Female gender, *n* (%)	25 (71)	9 (64)	16 (76)
DAS at baseline, median (IQR)	4.0 (3.3–4.6)	4.0 (3.7–4.6)	4.0 (3.3–4.5)
DAS at T26, median (IQR)	1.7 (0.8–2.1)	1.2 (0.4–2.0)	1.8 (1.0–2.4)
DAS at T26 ≤ 2.4, *n* (%)	28 (80)	12 (86)	16 (76)

*IQR, interquartile range*.

### Dynamics of the IFN Response During Immunosuppressive Therapy

In order to gain insight into the dynamics of the IFN response during COBRA and COBRA-light therapy, we first analyzed the expression of 6 IRGs at baseline (T0) and after 4 weeks (T4) and 13 weeks (T13) of treatment in the complete group of COBRA and COBRA-light combined.

**Figure 1:**
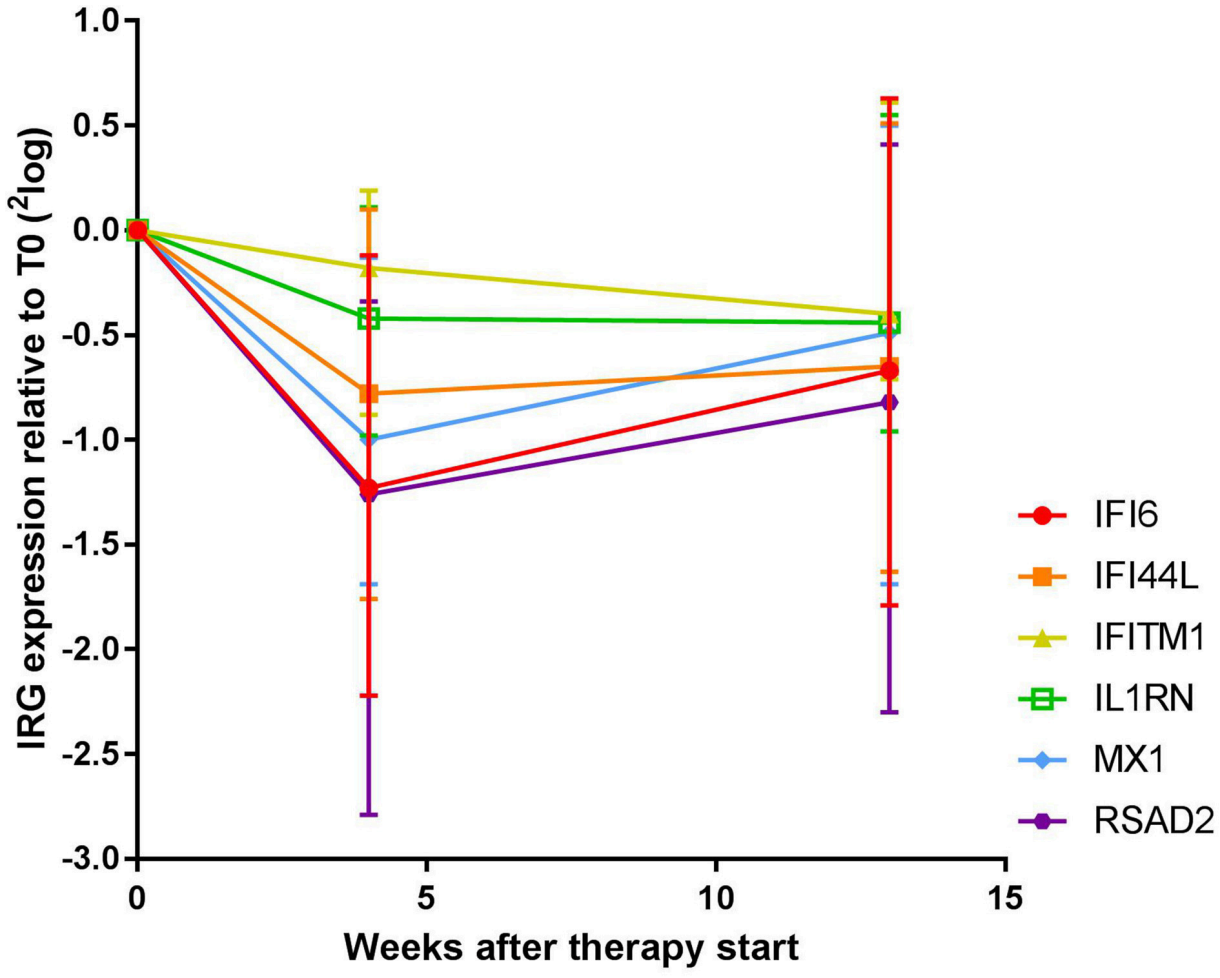
Expression dynamics of individual IRGs during COBRA and COBRA-light therapy. Both cohorts were merged for initial analysis.

As shown Figure 1 and Supplementary Table 1, expression of all measured IRGs except *IFITM1* and *IL1RN* displayed significant changes over all time points (Friedman test, *p* ≤ 0.016, vs. *p* ≥ 0.057 for *IFITM1* and *IL1RN*). These changes were most pronounced at T4, with median fold changes ranging from only 1.1-fold and 1.3-fold for *IFITM1* and *IL1RN*, respectively, up to 2.5-fold for *RSAD2* (Supplementary Figure 1A). In the significant genes, i.e., *IFI6, IFI44L, MX1*, and *RSAD2*, 69–77% of patients displayed a more than 1.2-fold decrease, whereas only 46 and 57% of the patients showed a more than 1.2-fold decrease in *IFITM1* and *IL1RN*, respectively (Supplementary Table 1). As displayed in Supplementary Figure 2, the extent of the fold change of T4 and T0 was partly dependent on the gene expression levels at baseline, i.e., higher baseline expression generally led to higher fold decreases. However, several patients displayed relatively low baseline expression and relatively high fold changes and vice versa, indicating that the extent of the fold change could not be fully explained by the baseline expression values.

Between T4 and T13, changes in IRG expression were either non-significant or displayed a moderate increase at the group level (1.0 to 1.4–fold increase, *p* = 0.012–0.29), indicating stabilization or even reversal of the IRG decrease that occurred after 4 weeks of treatment. Of note, overall dynamics were largely variable between patients (see Supplementary Figure 1B). Individual dynamics over time are displayed in Supplementary Figure 3.

### Relation Between Transcription Factor Binding Sites and Sensitivity to Immunosuppressive Downregulation

**Figure 2:**
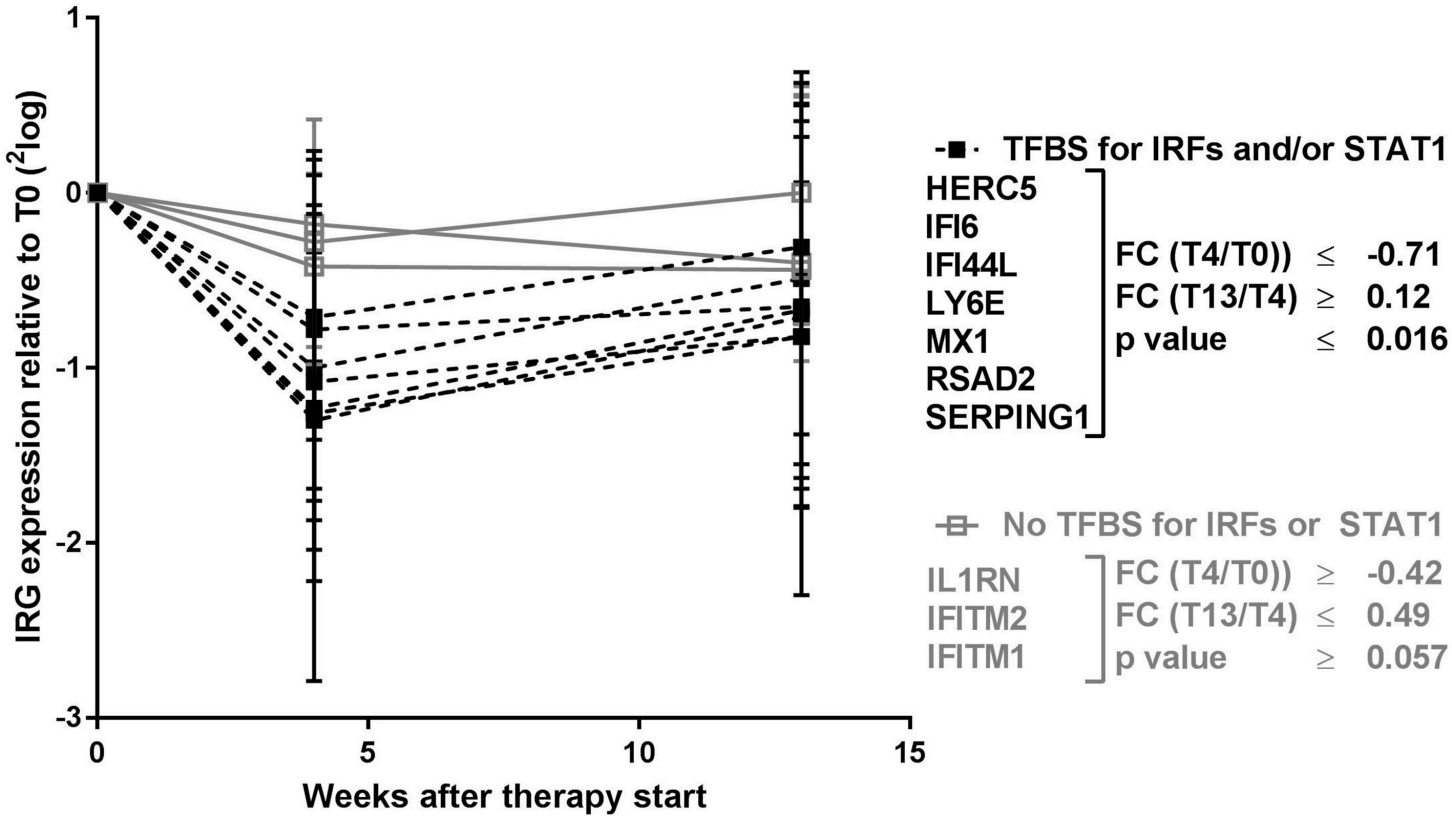
Expression dynamics of individual IRGs during COBRA and COBRA-light therapy. IRGs were categorized based on the presence or absence of transcription factor binding sites (TFBS) for IRF proteins and/or STAT1. FC, fold change expressed in ^2^log values. *P*-values are indicated for longitudinal analysis by Friedman test.

Remarkably, the two genes that appeared least affected by the COBRA and COBRA-light therapy, *IFITM1 and IL1RN*, both lacked binding sites for IRF-transcription factors and STAT1 (see Table 1). This implies that the therapy-related IRG reduction might be IRF-dependent and/or STAT1-dependent. In order to test this hypothesis, an additional selection of IRGs was made, based on the presence of binding sites for either IRF or STAT1 (see Table 1). As shown in Figure 2 and Supplementary Table 2, the additional IRG that lacked a TFBS for IRF proteins or STAT1, i.e*., IFITM2* displayed only moderate changes upon treatment, similar to *IL1RN* and *IFITM1* (*p* = 0.49). Accordingly, the additional genes with a TFBS for IRF proteins and/or STAT1 showed a considerable downregulation at the group level (*p* ≤ 0.012). This further suggests that the therapy-related IRG reduction is largely IRF- and STAT1-dependent.

### Differences in Dynamics of IFN Response Between COBRA and COBRA-Light Therapy

Since the main difference between COBRA and COBRA-light therapy is the dose of prednisolone and the use of SSZ, and previous studies have shown a potential suppressing effect of those two agents on IRG expression (7, 13, 15), we next analyzed the two therapy groups separately. The 7 IRGs with most distinct dynamics over time (*HERC5, IFI6, IFI44L, LY6E, MX1, RSAD2*, and *SERPING1*) were highly correlating (Spearman *r* ≥ 0.53, *p* < 0.001), hence expression levels of these genes were averaged into a 7-IRG score for visualization purposes.

**Figure 3:**
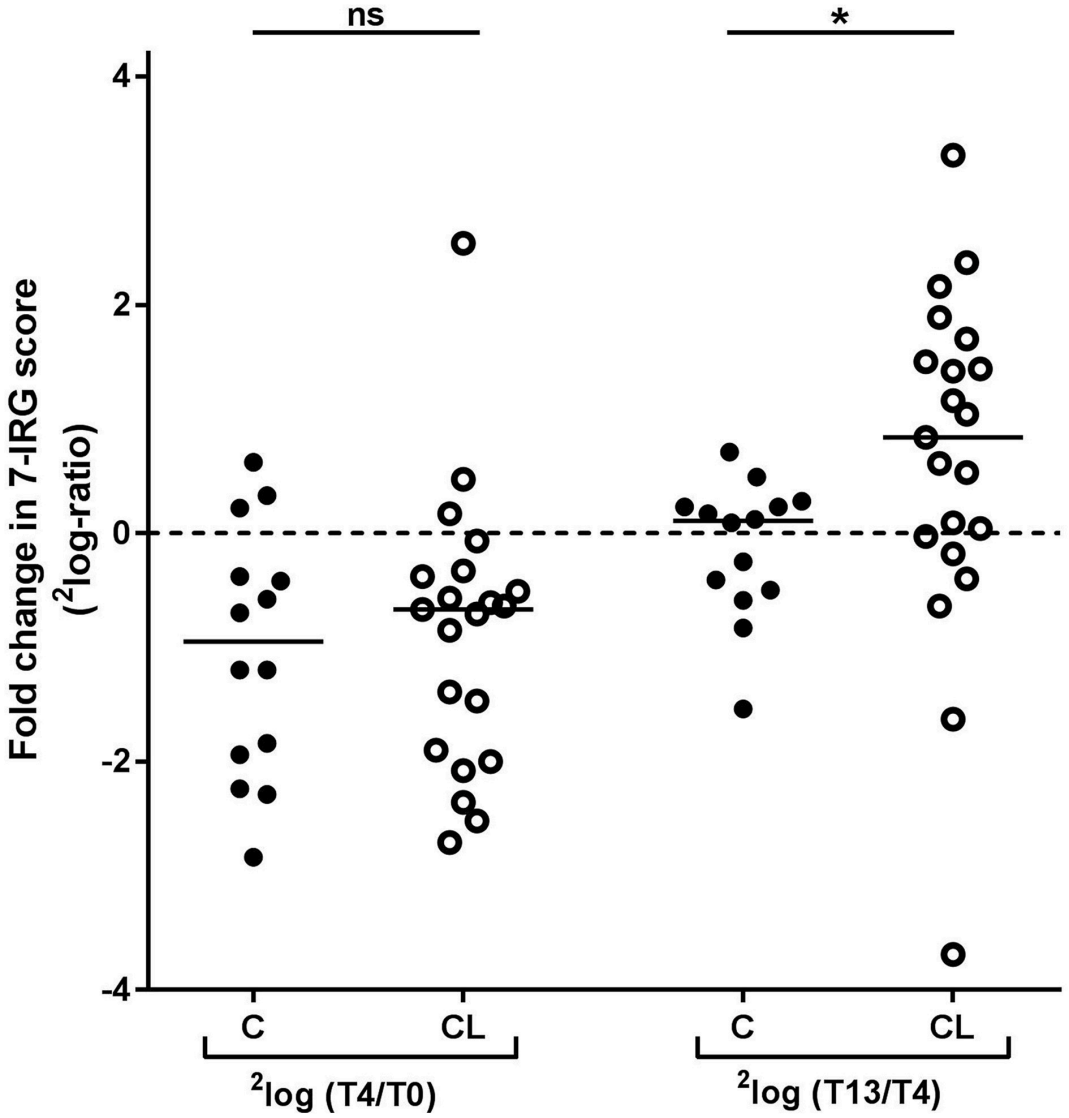
Comparison of longitudinal changes in 7-IRG score between COBRA (C) and COBRA-light(CL)-treated RA patients. ^*^p < 0.05.

As shown in Figure 3, both the COBRA and the COBRA-light group displayed a similar median decrease in IRG expression between T0 and T4, despite the difference in prednisolone dose and SSZ use (Comparison of fold changes, *p* ≥ 0.19). However, IRG dynamics between T4 and T13 appeared strikingly different; whereas in the COBRA-treated group IRG expression displayed only minor changes (median 1.1-fold, maximum 1.6-fold increase), the majority of the patients in the COBRA-light-treated group displayed an increase in expression (median 1.8-fold, up to maximum 9.9-fold.Comparison of fold changes in 7-IRG score *p* = 0.029). Significantly more COBRA-light-treated patients displayed an increase of at least 1.2-fold (chi-square *p* = 0.019). Similar results were found for the individual IRGs (Supplementary Figure 4). There was no significant correlation between T13/T4 ratio and baseline IRG expression in these groups (*p* ≥ 0.12, data not shown), indicating that these dynamics are dependent on the treatment rather than on the baseline expression levels.

### Dynamics of IFN Response in Relation to Clinical Response to Therapy

Despite the significant changes in the IFN response observed at the group level, we also observed substantial variation in IRG expression between individuals. For example, some patients did not display downregulation in any of the IRGs between T0 and T4, or only in a part of them (data not shown). Therefore, we also investigated whether these inter-individual variations could be related to the clinical response to COBRA and COBRA-light therapy.

Non-response was defined as DAS > 2.4 at T26. As such, the merged cohort consisted of 7 non-responders and 28 responders. Due to low numbers, the two cohorts could not be analyzed separately. In line with previous reports, no correlation was observed between baseline DAS and IRG expression (15, 17) (data not shown). As shown in Table 3 and Supplementary Table 3, no significant differences in the 7-IRG score or any of the treatment-sensitive IRGs were observed between responders and non-responders, at baseline nor in the expression and dynamics after 4 and 13 weeks (*p* ≥ 0.059). Furthermore, no significant correlation was observed between IRG expression and dynamics and the change in DAS after 13 and 26 weeks (unadjusted *p*-values ≥ 0.045).

**Table 3:** Assessment of 7-IRG score values and dynamics in relation to clinical response to COBRA and COBRA-light therapy.

	**7-IRG score at time point**			^****2****^**log-ratios in 7-IRG score**	
	**T0**	**T4**	**T13**	**T4/T0**	**T13/T4**
R vs. NR (DAS ≤2.4 or >2.4 at T26)	0.17	0.23	0.56	0.86	0.53
ΔDAS at T13 (correlation)	0.18	0.21	0.72	0.43	0.29
ΔDAS at T26 (correlation)	0.70	0.32	0.56	0.58	0.93
^2^Log-ratio CRP (T4/T0)	0.31	0.34	0.81	0.010(+)	0.22
^2^Log-ratio CRP (T13/T0)	0.087	0.68	0.54	0.066	0.25
^2^Log-ratio CRP (T26/T0)	0.12	0.17	0.30	0.61	0.90
^2^Log-ratio ESR (T4/T0)	0.23	0.49	0.68	0.013(+)	0.84
^2^Log-ratio ESR (T13/T0)	0.083	0.85	0.55	0.038(+)	0.36
^2^Log-ratio ESR (T26/T0)	0.063	0.36	0.63	0.16	0.75

*Table indicates p values. Details of the statistical analyses are described in the methods section. The direction of the significant correlations is indicated between brackets*.

At T4, where the maximum IRG decline was observed, DAS was not determined. Instead, we investigated CRP and ESR at T4 and later time points as indicators of inflammation. Interestingly, a significant positive correlation was observed between the change in IRG expression and change in both CRP and ESR between T0 and T4 (*p* ≤ 0.051, Pearson *r* ≥ 0.42 for 7-IRG score, see Table 3 and see Supplementary Tables 4, 5 for the individual IRGs). However, this correlation was diminished at later time points, suggesting that there is no relation with the eventual clinical response to COBRA and COBRA-light therapy. Separate analysis of the COBRA and COBRA-light group revealed similar results (data not shown).

## Discussion

In previous studies using cross-sectional data from RA patients, we observed lower IRG expression in patients using GCs, SSZ and hydroxychloroquine, but not in patients using MTX (7, 15). The unique and virtually complete longitudinal collection of PAXgene blood enabled us to investigate the influence of immunosuppressive therapy on the IFN response in a longitudinal setting. To our knowledge, the present study is the first to do so.

Using blood collected at baseline and after 4 and 13 weeks of COBRA or COBRA-light treatment, we observed a substantial downregulation of the IFN response within 4 weeks of therapy. This reduction was irrespective of the therapy group, but was not equally strong for each IRG. Between 4 and 13 weeks, however, IRG expression changes were highly variable between patients, which appeared partly dependent on the treatment.

The extent of the downregulation after 4 weeks of treatment was similar between COBRA and COBRA-light-treated patients. Most probably, this decline is due to the prednisolone treatment, as its dose is relatively high in both groups, and it acts more rapidly than MTX and SSZ. The absence of differences between COBRA and COBRA-light treatment at this time point suggests that prednisolone dose of 30 mg/day prednisolone already causes maximum downregulation. The expression dynamics seemed to be restricted to IRGs that contained one or more binding sites for IRF transcription factors and/or STAT1. Conversely, three genes that lacked such binding sites, displayed considerably less downregulation during treatment. Previous *in vitro* studies have shown that the GC signaling pathway, which is activated by prednisolone, is able to compete with the IFN signaling pathway for certain IRF proteins (12, 13) and to inhibit STAT1 activation (14), which could explain our observations.

Between T4 and T13, the IRG dynamics were more variable and differed between the two patient groups. Whereas normalization of IRG expression toward baseline levels was observed in the COBRA-light-treated group, IRG expression remained rather stable in the COBRA-treated group. This is particularly remarkable, as the prednisolone dose is equal between both groups at after 12 weeks (7.5 mg/day), and the only difference is the MTX dose (7.5 mg/week in COBRA and 25 mg/week in COBRA-light) and the addition of 2 g SSZ in the COBRA-treated groups. The total received dose of prednisolone, however, is 1.5-fold higher in the COBRA-treated group at this point. Possibly, the combination of SSZ and higher total prednisolone dose causes a more prolonged downregulation of the IFN response in the COBRA group. However, due to the combination of agents, it is not possible to strongly conclude which agent is responsible for the observed differences in dynamics.

Unfortunately, no untreated control-group with longitudinal follow-up was available, hence it cannot be fully excluded that the IRG dynamics we observed were a consequence of natural fluctuation. However, the correspondence with previously published *in vitro* data (12, 13) as well as our previous *in vivo* data (7, 15) and the observed differences between COBRA and COBRA-light strongly suggest that the observed changes in IRG expression are not spontaneous but truly mediated by the treatment.

The observation that not all IRGs appeared equally sensitive to the immunosuppressive agents of COBRA and COBRA-light therapies, and the putative influence of total prednisolone dose, could particularly be important when using the IFN response as a biomarker, which has been described for several biologics, including TNF inhibitors, rituximab, and tocilizumab (8, 10, 11, 19, 20). For example, we have demonstrated that the predictive performance of the 8-IRG geneset for non-response to rituximab is reduced when patients use prednisolone at the moment of blood collection, presumably because of a prednisolone-mediated reduction in IRG expression (7). Correspondingly, for 5 of the 8 genes in this geneset we have now shown that they indeed are sensitive to immunosuppressive treatment, including prednisolone.

Remarkably, the observation that the IRG downregulation attenuated in COBRA-light-treated patients implies that the IFN response could normalize upon reduction of the prednisolone dose. Hence, the 8-IRG geneset might still be applicable as a predictor for rituximab in patients who are tapering their prednisolone.

Moreover, it would be particularly interesting to investigate whether the IRGs that were less affected by COBRA and COBRA-light treatment could serve as alternative predictors for the response to biologics, since they do reflect IFN activity in RA (17), hence they might still play a role in the response to biologics. Interestingly, the gene *IFITM1*, which appeared less sensitive to prednisolone interference, has already been described as a predictor of rituximab nonresponse in a transcriptomics study (21). Alternatively, one study demonstrated an association between IFN-related gene variants and the response to rituximab (22). Although the predictive value was rather low, the concept of using IFN-related gene variants, which are naturally insensitive to therapy interference, would be interesting to study in further detail and with more IFN-related SNPs (23).

Besides the differential sensitivities of individual IRGs to the treatments, we also observed high heterogeneity in the IRG dynamics between patients. As described before, IRG expression in RA patients is generally highly heterogeneous, which we observed both at baseline and upon therapy. Although we observed a linear relation between baseline IRG expression and the extent of the downregulation after 4 weeks, the variation in IRG dynamics could not be fully explained by the baseline variation in IRG expression. This indicates that besides the type of treatment and the administered doses of treatment, there are also other factors that could influence the IFN response in RA. It has been well-discussed that the IRG response in RA patients is the result of several factors combined, such as extracellular stimuli (24), receptor expression (25) and genetic variation in signaling proteins (22, 23, 26). Considering the putative mechanism of IRG downregulation by prednisolone as described above, particularly the variation in signaling proteins could also contribute to a patient's sensitivity to the observed IRG downregulation. In addition, many other factors, independent of baseline IRG expression, such as therapy adherence and the patient's sensitivity to glucocorticoids (27) could hypothetically affect the extent of the IRG downregulation.

Despite this heterogeneity in the IFN response between patients, we did not observe an association between the IRG expression or dynamics and the response to COBRA and COBRA-light therapy. Considering the differences in IRG dynamics between COBRA and COBRA-light, the potential relation between IRG expression and clinical response should ideally be analyzed for both treatment groups separately. Since methotrexate has no proven interference with IRG expression, while prednisolone and sulfasalazine have, the use of IRG-interfering agents is considerably higher with COBRA-treatment compared to COBRA-light treatment. Moreover, as all agents have different modes of action (28–30), hence clinical response for each agent is probably achieved via different mechanisms. Consequently, it is possible that the relation between IRG expression and clinical response is different between COBRA and COBRA-light. Unfortunately, the current cohorts were too small to study this in detail.

Since DAS information was not available at T4, a direct comparison of DAS dynamics and IRG dynamics could not be made. Instead, we additionally investigated CRP and ESR as indicators of changes in inflammation in relation to IRG dynamics. Interestingly, a significant correlation was observed between IRG decline and CRP and ESR decline at T4, but not at later time points. At this early time point, clinical effects are mostly attributed to the prednisolone treatment, whereas at later time points more influence is anticipated from MTX and SSZ. As a consequence, the IRG dynamics at T4 could reflect the initial clinical response to prednisolone, but it does not predict the eventual clinical response as this is the result of the combination of agents. It would be interesting to study the potential relation between IRG dynamics and clinical response in patients using prednisone as monotherapy compared to patients using MTX and/or SSZ monotherapy.

In summary, we have demonstrated that both COBRA and COBRA-light therapy are able to downregulate the IFN response in RA. The dynamics of this downregulation were partly dependent on the presence of TFBS within the IRGs and the combination and dosages of agents, but they were irrespective of the clinical response to therapy. Altogether, these results shed a new light on the behavior of the IFN response in RA.

## Ethics Statement

This study was approved by the medical ethics committee of VU University Medical Center and Reade, Amsterdam, The Netherlands, and informed consent was obtained from all donors.

## Author Contributions

All authors were involved in drafting the article or revising it critically for important intellectual content, and all authors approved the final version to be published. TdJ and WL: study concept and design. TdJ, TS, EM, and WL: acquisition of patient material and data. TdJ, TS, EM, CvdL, RvV, and WL: analysis and interpretation of data.

### Conflict of Interest Statement

RvV reports grants and research support from AbbVie, Amgen, Bristol-Myers Squibb, GSK, Lilly, Pfizer, Roche, and UCB Pharma and personal fees from AbbVie, Biotest, Bristol-Myers Squibb, Celgene, Crescendo, GSK, Janssen, Lilly, Novartis, Pfizer, Roche, and UCB pharma, outside the submitted work. The remaining authors declare that the research was conducted in the absence of any commercial or financial relationships that could be construed as a potential conflict of interest.
