# Supplementary material for: Dynamics of the Type I Interferon Response During Immunosuppressive Therapy in Rheumatoid Arthritis
Source: Front Immunol. 2019 Apr 24;10:902. doi: 10.3389/fimmu.2019.00902 (PMC6491736; doi:10.3389/fimmu.2019.00902)
Supplement: Supplementary file 1 [file Data_Sheet_1.pdf]

Supplementary Material

Dynamics of the Type I Interferon Response During Immunosuppressive Therapy in Rheumatoid Arthritis

Tamarah D. de Jong[1]*, Tanja Snoek[1], Elise Mantel[1], Conny J. van der Laken[1], Ronald F. van Vollenhoven[1], Willem F. Lems[1]

[1]Amsterdam UMC, Vrije Universiteit Amsterdam, Rheumatology, Amsterdam Rheumatology and immunology Center, De Boelelaan 1117, Amsterdam, the Netherlands

*Correspondence:

Dr. Tamarah de Jong: tamarahdesiree@gmail.com

1 Supplementary Tables

Supplementary Table 1 Dynamics of the IFN response at different time points during COBRA and COBRA-light therapy.

		IFI6	IFI44L	IFITM1	IL1RN	MX1	RSAD2
All time points	P value	<0.001	0.016	0.36	0.057	0.003	<0.001
T4 vs. T0	Median FC	-1.2	-0.78	-0.18	-0.42	-1.0	-1.3
	FC range	(-3.0-1.8)	(-3.2-2.7)	(-2.0-1.1)	(-2.4-1.4)	(-3.4-2.1)	(-4.1-3.8)
	FC < -0.26 (%)	71	69	46	57	74	77
	P value	<0.001	<0.001	0.049	0.0084	<0.001	<0.001
T13 vs. T4	Median FC	0.47	0.32	0.25	0.029	0.16	0.45
	FC range	(-1.8-4.3)	(-3.8-4.1)	(-1.6-2.5)	(-2.2-2.0)	(-3.7-3.2)	(-4.9-4.9)
	FC > 0.26 (%)	57	51	49	46	49	63
	P value	0.035	0.12	0.29	0.17	0.17	0.025
T13 vs. T0	Median FC	-0.67	-0.65	-0.40	-0.44	-0.49	-0.82
	FC range	-3.2-3.0	-3.3-3.3	-2.8-2.1	-2.9-2.7	-4.4-4.2	-3.0-1.7
	FC < -0.263 (%)	60	57	57	54	63	63
	P value	0.024	0.055	0.39	0.16	0.029	0.018

FC, fold change, displayed as ^{2}log-ratios. A FC< -0.26 and > 0.26 are the ^{2}log ratio equivalents to a 1.2-fold decrease and increase, respectively.

Supplementary Table 2 Dynamics of the additionally measured IRGs at different time points during COBRA and COBRA-light therapy.

		HERC5	IFITM2	LY6E	SERPING 1
All time points	P value	0.012	0.49	0.015	<0.001
T4 vs. T0	Median FC	-1.1	-0.28	-0.71	-1.3
	FC range	-3.6-3.3	-2.3-1.6	-4.8-2.8	-4.1-2.4
	FC < -0.26 (%)	69	51	60	74
	P value	0.0020	0.13	0.0059	<0.001
T13 vs. T4	Median FC	0.24	0.034	0.12	0.18
	FC range	-4.5-3.3	-1.7-2.3	-4.0-2.4	-3.2-4.5
	FC > 0.26 (%)	49	43	46	49
	P value	0.27	0.50	0.51	0.077
T13 vs. T0	Median FC	-0.82	-0.0036	-0.31	-0.71
	FC range	-3.7-2.6	-2.5-1.4	-5.0-2.7	-4.8-4.1
	FC < -0.26 (%)	60	43	57	63
	P value	0.029	0.58	0.029	0.027

FC, fold change, displayed as ^{2}log-ratios. A FC< -0.26 and > 0.26 are the ^{2}log ratio equivalents to a 1.2-fold decrease and increase, respectively.

Supplementary Table 3 Unadjusted P values of assessing IRG expression and IRG dynamics in relation to clinical response.

	T0	T4	T13	T4/T0	T13/T4
**Responders vs. non-responders (DAS ≤2.4 or >2.4 after 26 weeks)**					
	**T0**	**T4**	**T13**	**T4/T0**	**T13/T4**
HERC5	0.32	0.41	0.33	1.0	0.92
IFI44L	0.095	0.17	0.61	0.67	0.51
IFI6	0.36	0.23	0.94	0.70	0.059
LY6E	0.28	0.059	0.10	0.25	0.59
MX1	0.11	0.45	0.88	0.18	0.36
RSAD2	0.20	0.59	0.61	0.43	0.28
SERPING	0.89	0.76	0.64	0.89	0.92
**Correlation with ΔDAS at 13 weeks**					
	**T0**	**T4**	**T13**	**T4/T0**	**T13/T4**
HERC5	0.16	0.55	0.92	0.29	0.59
IFI44L	0.47	0.069	0.81	0.75	0.045
IFI6	0.39	0.28	0.52	0.56	0.075
LY6E	0.10	0.76	0.77	0.16	0.79
MX1	0.29	0.13	0.90	0.62	0.24
RSAD2	0.15	0.086	0.58	0.85	0.089
SERPING	0.49	0.98	0.45	0.33	0.51
**Correlation with ΔDAS at 26 weeks**					
	**T0**	**T4**	**T13**	**T4/T0**	**T13/T4**
HERC5	0.37	0.27	0.27	0.90	0.93
IFI44L	0.81	0.20	0.66	0.19	0.54
IFI6	0.96	0.46	0.91	0.77	0.48
LY6E	0.35	0.33	0.17	0.81	0.79
MX1	0.73	0.32	0.41	0.88	0.87
RSAD2	0.65	0.32	0.86	0.77	0.58
SERPING	0.96	0.82	0.92	0.83	0.79

Supplementary Table 4 Unadjusted P values of assessing IRG expression and IRG dynamics in relation to CRP dynamics.

**Correlation with CRP log-ratio T4/T0**					
	**T0**	**T4**	**T13**	**T4/T0**	**T13/T4**
HERC5	0.45	0.20	0.43	0.026 (+)	0.034 (-)
IFI44L	0.25	0.68	0.69	0.023 (+)	0.34
IFI6	0.083	0.64	0.90	0.062	0.55
LY6E	0.90	0.31	0.62	0.19	0.092
MX1	0.26	0.52	0.68	0.016 (+)	0.29
RSAD2	0.21	0.46	0.93	0.017 (+)	0.38
SERPING1	0.89	0.049	0.63	0.049 (+)	0.19
**Correlation with CRP log-ratio T13/T0**					
	**T0**	**T4**	**T13**	**T4/T0**	**T13/T4**
HERC5	0.12	0.90	0.99	0.069	0.90
IFI44L	0.097	0.34	0.57	0.20	0.079
IFI6	0.045 (-)	0.24	0.36	0.14	0.022 (+)
LY6E	0.22	0.90	0.89	0.096	0.98
MX1	0.096	0.22	0.87	0.33	0.36
RSAD2	0.078	0.57	0.39	0.14	0.099
SERPING1	0.34	0.46	0.12	0.12	0.42
**Correlation with CRP log-ratio T26/T0**					
	**T0**	**T4**	**T13**	**T4/T0**	**T13/T4**
HERC5	0.24	0.16	0.15	0.86	0.92
IFI44L	0.12	0.21	0.33	0.45	0.96
IFI6	0.050 (-)	0.19	0.60	0.20	0.55
LY6E	0.33	0.27	0.21	0.95	0.89
MX1	0.060	0.17	0.16	0.27	0.68
RSAD2	0.11	0.22	0.46	0.56	0.72
SERPING1	0.48	0.39	0.63	0.86	0.74

For the p values < 0.05, absolute Pearson correlation coefficients ranged from 0.34 – 0.43. Directions of the significant correlations are indicated by (-) or (+).

Supplementary Table 5 Unadjusted P values of assessing IRG expression and IRG dynamics in relation to ESR dynamics.

**Correlation with ESR log-ratio T4/T0**					
	**T0**	**T4**	**T13**	**T4/T0**	**T13/T4**
HERC5	0.25	0.38	0.98	0.027 (+)	0.36
IFI44L	0.32	0.77	0.78	0.076	0.96
IFI6	0.17	0.90	0.59	0.047 (+)	0.61
LY6E	0.50	0.57	0.98	0.13	0.54
MX1	0.28	0.92	0.79	0.12	0.83
RSAD2	0.18	0.58	0.59	0.028 (+)	0.96
SERPING1	0.29	0.12	0.31	0.010 (+)	0.63
**Correlation with ESR log-ratio T13/T0**					
	**T0**	**T4**	**T13**	**T4/T0**	**T13/T4**
HERC5	0.072	0.89	0.80	0.034 (+)	0.90
IFI44L	0.19	0.59	0.62	0.22	0.23
IFI6	0.16	0.68	0.56	0.15	0.27
LY6E	0.13	0.91	0.97	0.082	0.93
MX1	0.16	0.47	0.85	0.26	0.39
RSAD2	0.084	0.77	0.44	0.092	0.22
SERPING1	0.064	0.56	0.11	0.025 (+)	0.32
**Correlation with ESR log-ratio T26/T0**					
	**T0**	**T4**	**T13**	**T4/T0**	**T13/T4**
HERC5	0.040 (-)	0.29	0.50	0.29	0.74
IFI44L	0.12	0.49	0.72	0.16	0.80
IFI6	0.18	0.72	0.88	0.15	0.88
LY6E	0.098	0.20	0.24	0.58	0.90
MX1	0.056	0.39	0.47	0.11	0.94
RSAD2	0.089	0.58	0.89	0.17	0.67
SERPING1	0.10	0.43	0.92	0.49	0.53

For the p values < 0.05, absolute Pearson correlation coefficients ranged from 0.34 – 0.44. Directions of the significant correlations are indicated by (-) or (+).

2 Supplementary Figures

A

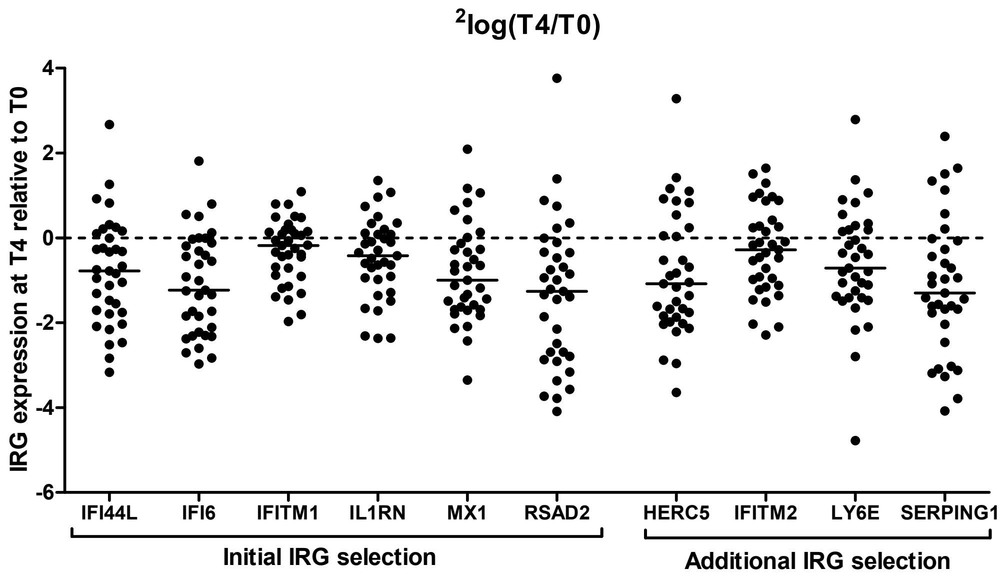

B

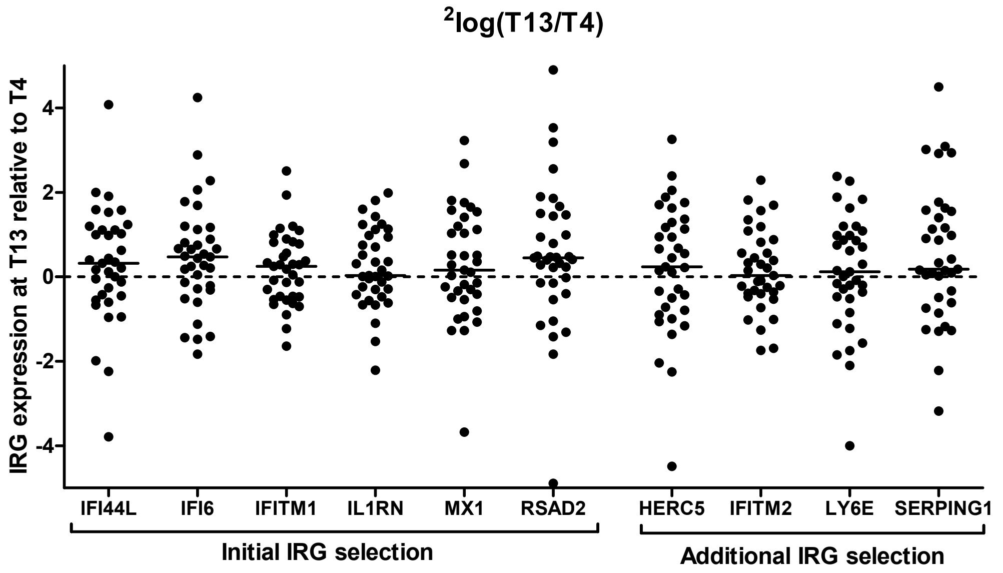

Supplementary Figure 1 Log-fold changes in IRG expression between baseline (T0) and 4 weeks (T4) and between 13 weeks (T13) and 4 weeks, displayed per IRG.

A

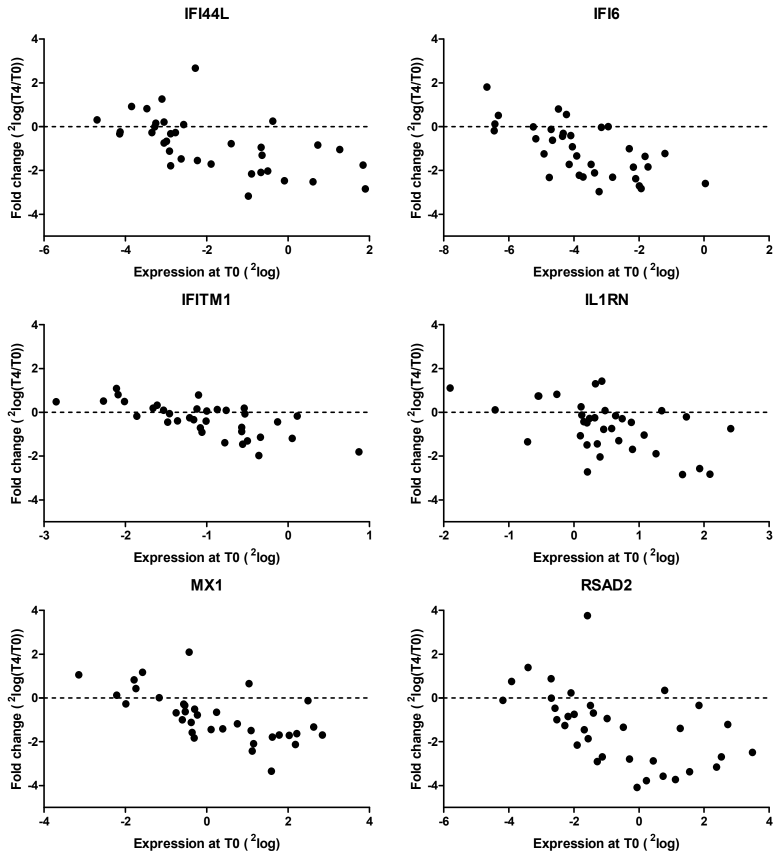

B

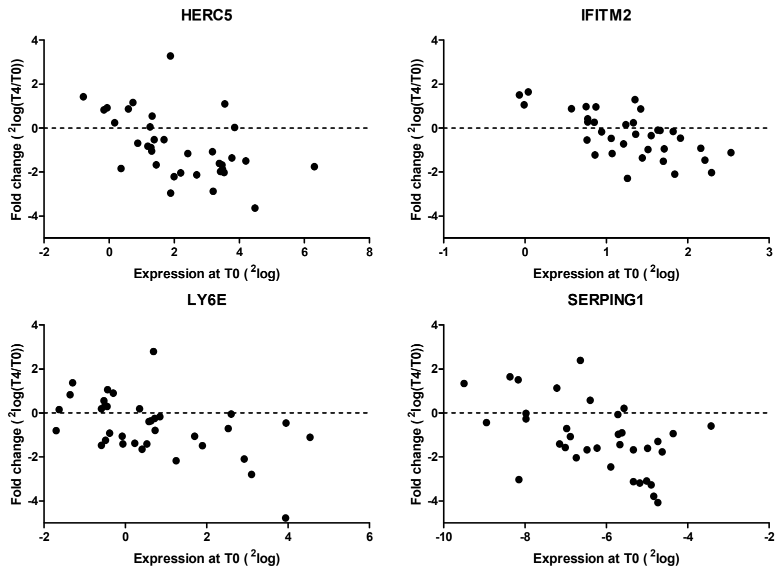

Supplementary Figure 2 Correlations between the fold change in IRG expression at 4 weeks (^{2}log(T4/T0)) and the initial IRG expression at baseline. A) initially selected IRGs and B) additionally selected IRGs.

A

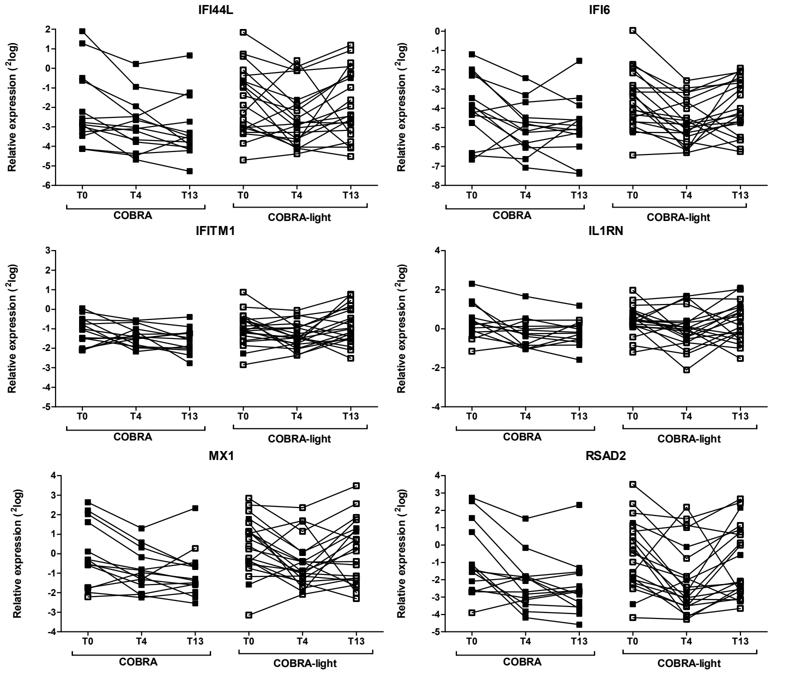

B

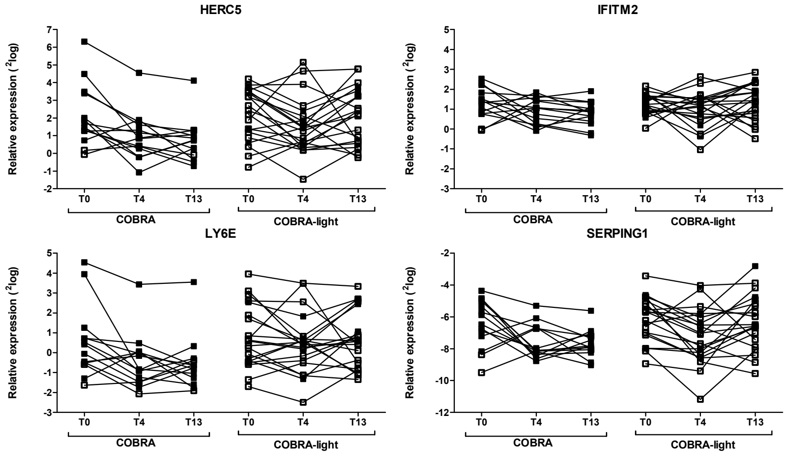

Supplementary Figure 3 Individual dynamics of IRG expression during COBRA and COBRA-light therapy. A) Initially selected IRGs, B) Additionally selected IRGs.

A

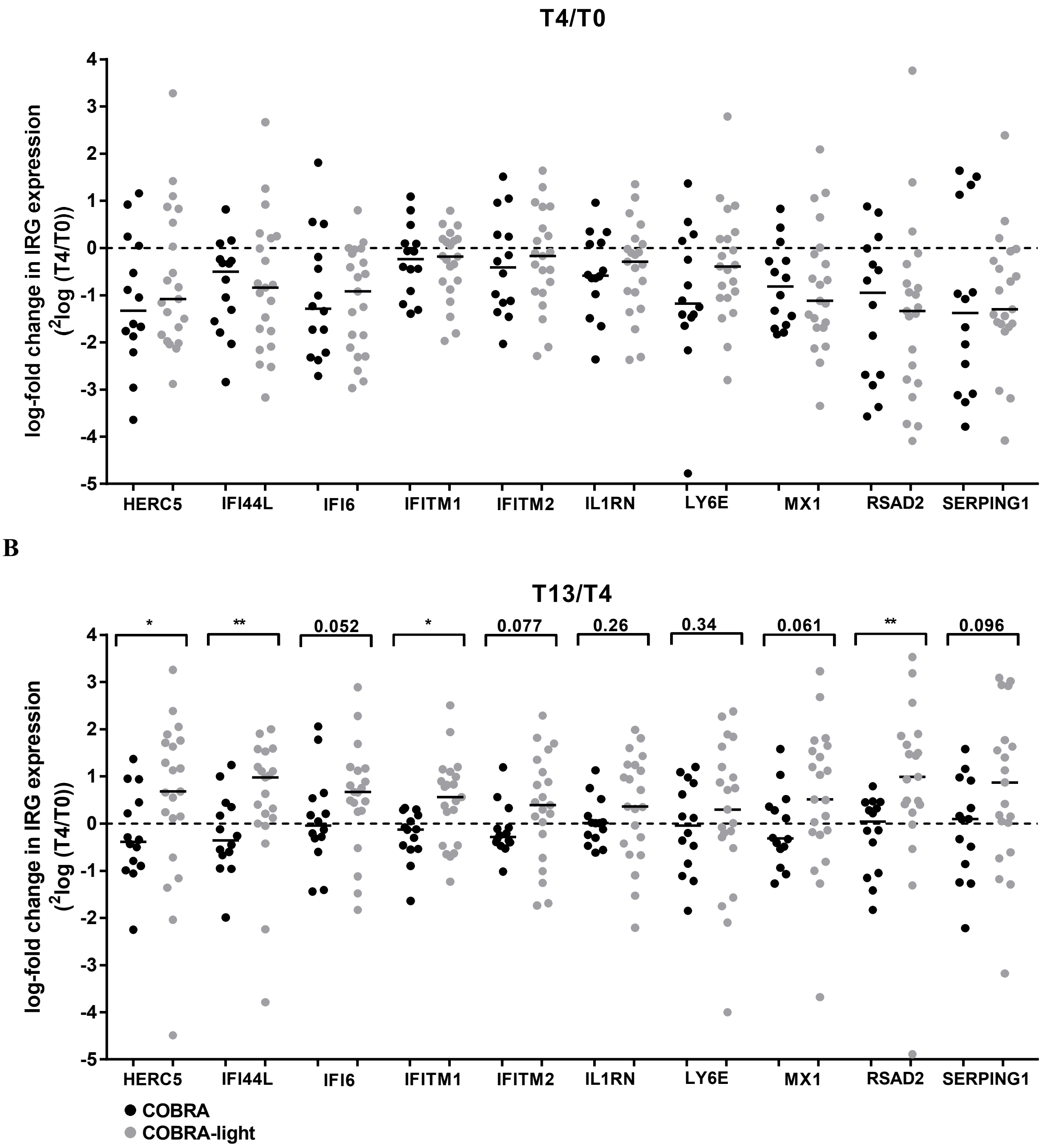

Supplementary Figure 4 Comparison of longitudinal changes in individual IRGs between COBRA (C) and COBRA-light(CL)-treated RA patients. A) ^{2}log-fold changes between T0 and T4. No significant differences were observed between COBRA and COBRA-light (p≥0.19) B) ^{2}log-fold changes between T4 and T13. Significance is indicated above the datasets. ** p<0.01, * p<0.05-0.01.
